# ^1^H, ^13^C and ^15^N resonance assignments for the fibrillin-1 EGF2-EGF3-hybrid1-cbEGF1 four-domain fragment

**DOI:** 10.1007/s12104-013-9481-7

**Published:** 2013-05-07

**Authors:** Ian B. Robertson, Isabelle Osuch, David A. Yadin, Penny A. Handford, Sacha A. Jensen, Christina Redfield

**Affiliations:** Department of Biochemistry, University of Oxford, South Parks Road, Oxford, OX1 3QU UK

**Keywords:** NMR assignment, Fibrillin, Epidermal growth factor-like (EGF), Microfibril, Calcium-binding, Hybrid domain

## Abstract

Fibrillins are large extracellular glycoproteins that form the principal component of microfibrils. These perform a vital structural function in the extracellular matrix of many tissues. Fibrillins have also been implicated in mediating a number of protein–protein interactions, some of which may be significant in regulating growth factors such as transforming growth factor β. Here we present the backbone and side-chain ^1^H, ^13^C and ^15^N assignments for a 19 kDa protein fragment derived from the N-terminus of human fibrillin-1, encompassing four domains in total. These domains include the second and third epidermal growth factor-like (EGF) domains, the first hybrid domain (hyb1), and the first calcium-binding EGF domain of fibrillin-1. This region of fibrillin-1 is of particular interest as the hyb1 domain has been suggested to play a role in microfibril assembly, as well as several other protein–protein interactions.

## Biological context

Fibrillins assemble into oligomeric microfibrils, 10–12 nm in diameter, with a “beads on a string” appearance when viewed by rotary shadowing electron microscopy (Handford 2000; Lu et al. 2006). However, the protein interactions that drive assembly of microfibrils are poorly understood. Pulse-chase studies of chick aortae demonstrated that, after secretion, fibrillin was rapidly incorporated into disulphide-bonded aggregates (Reinhardt et al. 2000). While the identity of the intermolecular disulphide bonds driving this aggregation remains unclear, it has been demonstrated that the fibrillin hyb1 domain contains an additional cysteine residue (Cys 204), which is present as a free thiol when expressed in cell culture (Reinhardt et al. 2000). This additional cysteine is also highly conserved (Robertson et al. 2011), and so presents a good candidate for intermolecular disulphide bond formation.

The hyb1 domain of fibrillin-1, and its flanking domains, have also been implicated in a number of protein–protein interactions, particularly interactions with latent transforming growth factor β (TGFβ) binding proteins (LTBPs) and fibulins (Isogai et al. 2003; Massam-Wu et al. 2010; Ono et al. 2009). The interaction with LTBPs is of particular interest, as it may sequester TGFβ to microfibrils, and could play a role in disease progression in Marfan syndrome (MFS), where fibrillin haploinsufficiency has been associated with over-activation of TGFβ (Neptune et al. 2003). Inhibiting TGFβ signalling in mouse models of MFS has been shown to restore near normal development in many tissues (Cohn et al. 2007; Habashi et al. 2006, 2011; Ng et al. 2004), demonstrating a biologically significant connection between fibrillin and TGFβ signalling.

**Fig. 1:**
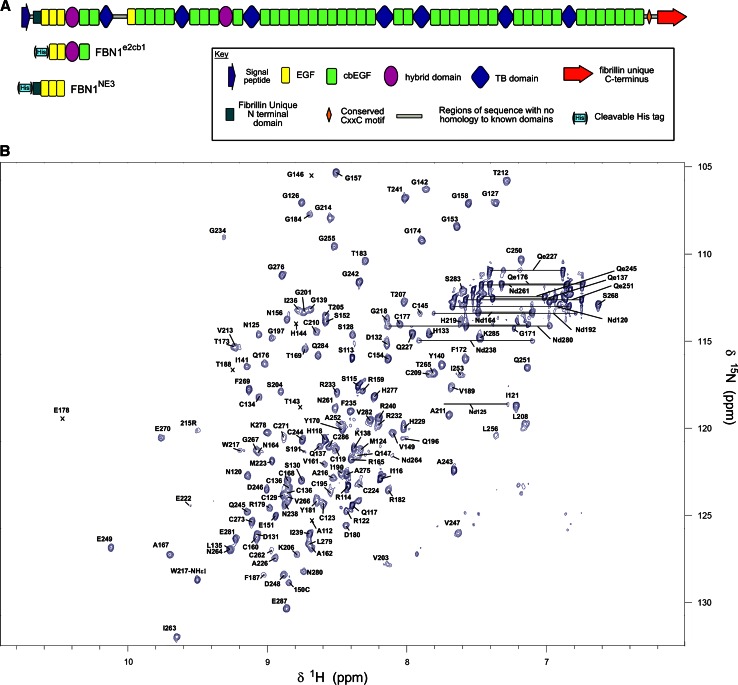
**a** Domain organisation of constructs FBN1^e2cb1^ and FBN1^NE3^, discussed in this study, and their size in relation to the full-length fibrillin molecule. **b** 950 MHz ^1^H-^15^N HSQC spectrum for FBN1^e2cb1^ in 95 % H_2_O/5 % D_2_O at pH 5.4 with 5 mM CaCl_2_. Peak assignments are shown above their respective peaks, except where otherwise indicated by *lines*. *Small crosses* indicate peaks that were too weak to visualise in this spectrum at the contour level selected

Here we present backbone and side-chain assignments for the EGF2-EGF3-hyb1-cbEGF1 domains of human fibrillin-1 (FBN1^e2cb1^) (Fig. 1a), derived from a protein fragment of 177 amino acids, from Ser 113 to Glu 287. These assignments will be used to investigate the structure and dynamics of this four-domain fibrillin-1 fragment, and also determine potential binding sites for LTBPs and other molecules.

## Methods and experiments

Protein expression and purification was carried out in a similar fashion to that described previously (Knott et al. 1996) but with modifications. The protein fragment was expressed in *Escherichia coli* BL21 cells transformed with a pQE-30 (Qiagen) expression vector and pREP4 plasmid for control of expression via the lac repressor. When cloned into the expression vector, an N-terminal His_6_ tag was included for purification, followed by a Ser-Ala spacer and a factor Xa protease recognition site (Ile-Glu-Gly-Arg) for later removal of the His_6_ tag. The additional cysteine in the hybrid domain (Cys 204) was also replaced with a serine as described previously (Jensen et al. 2009). This change was necessary to allow effective in vitro refolding of the protein fragment.

Protein was double labelled with ^15^N and ^13^C by growing cells in M9 medium containing 0.1 % (w/v) ^15^NH_4_Cl and 0.5 % (w/v) ^13^C-glucose (Goss Scientific), in the presence of 100 μg/ml ampicillin and 25 μg/ml kanamycin. 50 ml of starter culture, grown in unlabelled M9 medium at 37 °C for ~18 h, was used to inoculate 600 ml of labelled M9 medium. Bacteria were grown until OD_600_ reached ~0.8, at which point expression was induced with isopropyl-*β*-d-thiogalactopyranoside (IPTG) at a final concentration of 1 mM. Cells were then incubated at 28 °C for ~20 h, harvested by centrifugation and frozen at −80 °C prior to protein purification.

The expressed fibrillin protein appeared to form insoluble inclusion bodies, which did not dissolve in buffer containing 6 M guanidine, and so to increase protein yield an inclusion body extraction was carried out. The bacterial pellet was thawed and resuspended in a lysis buffer of 25 % (w/v) sucrose, 50 mM Tris–HCl pH 8.0, and 1 mM EDTA (20 ml buffer per litre of culture). Lysozyme was also added (2.5 mg per litre culture) to help break cell walls. Once resuspended the cells were lysed by sonication with 5 bursts of 30 s at 20 W (Jencons Ultrasonic Processor), swirling the mixture between bursts to make sure that the temperature did not increase locally. The lysate was then centrifuged at 12,000 rpm (JA-20 rotor) for 20 min. The resulting supernatant was discarded and the pellet was resuspended in inclusion body buffer 1 (25 ml per litre of culture), containing 1 % (w/v) sodium deoxycholate, 200 mM NaCl, 2 mM EGTA, and 20 mM Tris–HCl pH 8.0. Resuspension was assisted with repeated stirring and sonication. The mixture was then centrifuged again at 12,000 rpm (JA-20 rotor) for 20 min and the supernatant discarded. The pellet was resuspended in inclusion body buffer 2 (25 ml per litre of culture), containing 0.25 % sodium deoxycholate, 1 mM EGTA, and 20 mM Tris–HCl pH 8.0. Resuspension was assisted with repeated stirring and sonication. The mixture was centrifuged at 12,000 rpm (JA-20 rotor) for 20 min. Resuspension in inclusion body buffer 2 was repeated three times, or as many as necessary to remove the majority of white membrane residue and give a pure brown pellet. The inclusion body pellet was then dissolved in 8 M urea, with 0.1 mM NaN_3_, 1 mM EGTA, 50 mM dithiothreitol and 10 mM Tris–HCl pH 8.0, using a hand homogeniser to break up the inclusion bodies. The resulting protein-urea solution was then acidified and dialysed against 0.1 % (v/v) trifluoroacetic acid (TFA). After dialysis, SDS-PAGE was used to confirm the purity of the dissolved inclusion bodies. Protein solution was then transferred directly into a refold mix consisting of an aqueous solution of ~0.2 mg/ml reduced protein, 50 % (v/v) glycerol (to enhance protein solubility), 100 mM Tris–HCl pH 8.3, 3 mM cysteine and 0.3 mM cystine, and 50 mM CaCl_2_, which was then left for 48 h at 4 °C. After this period the refold mixture was acidified to pH 2 with HCl, and dialysed against 0.1 % (v/v) TFA overnight. Dialysate was centrifuged and filtered to remove any precipitate, concentrated by ultrafiltration, filtered again, and then purified by high performance liquid chromatography (HPLC) using a C8 reverse phase column (Rainin).

The His_6_ tag was cleaved off by incubation with factor Xa (Novagen), carried out with a maximum protein concentration of 1.5 mg/ml, with 30 mM MES-HCl pH 6, 100 mM NaCl, 10 mM CaCl_2_, and 1 unit factor Xa per mg protein, and incubated at 37 °C overnight. Protein was further purified by cation exchange fast protein liquid chromatography (FPLC) using a MonoS 5/50 GL column (GE Healthcare). For these purifications the buffers used were 30 mM citrate solution at pH 4, with 2 M NaCl added to the elution buffer. After FPLC all proteins were acidified to ~pH 2, filtered to remove any precipitate, and desalted by further HPLC purification before final lyophilisation. The final product was analysed by SDS-PAGE in the presence or absence of 5 % (v/v) 2-mercaptoethanol for reduction of disulphide bonds, as well as by electrospray ionisation mass spectrometry (observed mass = 18,820 Da).

NMR experiments for assignment were performed using ^15^N/^13^C double-labelled protein at a concentration of 1–1.5 mM in either 5 % D_2_O/95 % H_2_O (v/v) or 100 % D_2_O, with 5 mM CaCl_2_ at pH 5.4. NMR experiments were carried out at 25 °C on three different spectrometers: a home-built spectrometer with a ^1^H operating frequency of 950 MHz, a triple-resonance pulsed-field-gradient probe and GE/Omega data acquisition system, a second home-built spectrometer with a ^1^H operating frequency of 750 MHz, a triple-resonance pulsed-field-gradient probe and GE/Omega data acquisition system, or a Bruker Avance 500 MHz spectrometer with a Cryoplatform, equipped with a TCI CryoProbe. Backbone assignments were obtained using 3D HNCA, CBCANH, CBCA(CO)NH, HNCO, HN(CA)CO and HBHA(CO)NH triple-resonance experiments, and side-chain assignments were obtained with 3D HCCH-TOCSY (collected in 100 % D_2_O), 3D ^13^C-edited NOESY-HSQC (collected in both 100 % D_2_O and 5 % D_2_O/95 % H_2_O), and 2D ^1^H NOESY (collected in 100 % D_2_O) experiments. NMR data were processed using NMRPipe (Delaglio et al. 1995) and analysed using CcpNmr Analysis (Vranken et al. 2005).

## Assignment and data deposition

The FBN1^e2cb1^ fragment expressed contains 177 amino acids following factor Xa cleavage. Assignments were obtained for 88 % of all ^1^H resonances, 81 % of ^13^C resonances, and 69 % of ^15^N resonances. More specifically, 94 % of backbone ^1^H^N^, 87 % of backbone ^15^N, 93 % of Hα, 92 % of Hβ, 95 % of Cα, 94 % of Cβ and 93 % of C′ resonances were assigned. An annotated ^1^H-^15^N HSQC is shown in Fig. 1b. A significant number of residues gave extremely weak peaks, which prevented complete backbone assignment. Side-chain assignment was also hindered, to an extent, by weak or absent peaks, and also by strong peaks obscuring other resonances in some regions of 3D HCCH-TOCSY and 3D ^13^C-edited NOESY-HSQC spectra. ^15^N resonances from proline residues were not assigned.

**Fig. 2:**
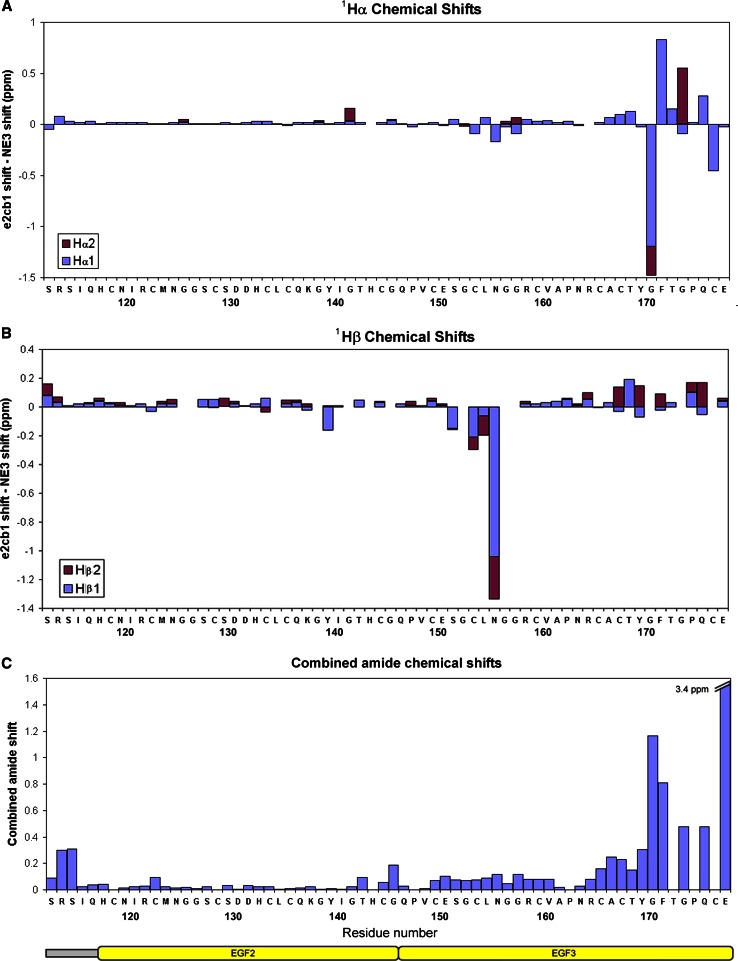
Chemical shift differences between FBN1^e2cb1^ and FBN1^NE3^. **a** Difference in Hα peak position (for glycines Hα1 corresponds to the more downfield resonance and Hα2 to the more upfield resonance). **b** Difference in Hβ peak position (Hβ1 corresponds to the more downfield resonance and Hβ2 to the more upfield resonance). **c** Combined amide shift difference, defined as [(Δ^1^H^N^)^2^ + (Δ^15^N/6)^2^]^1/2^

Assignments have been published previously for a fragment of the fibrillin N-terminus, including the fibrillin unique N-terminal FUN domain, EGF1, EGF2 and EGF3 (FBN1^NE3^) (Yadin et al. 2012). The EGF2 and EGF3 domains of this protein fragment overlap with the fragment assigned here (Fig. 1a), and comparison of the Hα, Hβ, and amide assignments for the two overlapping domains of both fragments demonstrates similar peak positions for many of the EGF2-EGF3 resonances (Fig. 2). This supports both domains adopting similar folds in the FBN1^NE3^ and FBN1^e2cb1^ constructs. However, significant changes in peak position were seen for some selected residues, especially for residues corresponding to the C-terminal loop of EGF3 (residues 171–178). In homologous EGF structures this region is involved in inter-domain packing interactions with a downstream domain (Downing et al. 1996; Jensen et al. 2012; Lee et al. 2004; Smallridge et al. 2003). For EGF3 this is the hyb1 domain, which is present in FBN1^e2cb1^, but absent from FBN1^NE3^. In EGF and cbEGF domains packing with the downstream domain is particularly centred around a glycine-aromatic residue pair in the C-terminal loop, and a Gly-Phe motif is found here in fibrillin-1 EGF3 (residues 171–172). The Hα resonances of Gly 171 exhibit a significant upfield shift in FBN1^e2cb1^ compared to FBN1^NE3^, which may be due to ring-current effects from packing with an aromatic side chain, induced by the presence of hyb1. The Hα resonance of Phe 172 on the other hand exhibits a downfield shift when hyb1 is present, which may also be influenced by packing interactions.

The Hβ protons of Asn 156 also demonstrate significant upfield shifts from 2.16 and 2.19 in FBN1^NE3^ to 1.12 and 1.89 ppm in FBN1^e2cb1^, suggestive of packing above or below the plane of an aromatic ring. While not at the C-terminus of the EGF3 sequence, this asparagine is predicted from homology modelling to be on a loop that would be close to the C-terminus of the EGF3 domain when folded, and could be brought into contact with aromatic residues at the C-terminus of EGF3 or in the hyb1 domain. Generally the differences in EGF3 chemical shifts between FBN1^NE3^ and FBN1^e2cb1^ strongly suggest domain interface formation between EGF3 and hyb1 in FBN1^e2cb1^, which has implications for the overall structure of the fibrillin molecule, and may also affect the presentation of protein interaction sites in this region.

The chemical shift assignments for fibrillin-1 EGF2-EGF3-hyb1-cbEGF1 have been deposited in the BioMagResBank (http://www.bmrb.wisc.edu) under the accession number 19078.
